# Endocrine and pubertal disturbances in optic nerve hypoplasia, from infancy to adolescence

**DOI:** 10.1186/s13633-015-0005-3

**Published:** 2015-04-15

**Authors:** Oliver J Oatman, Donald R McClellan, Micah L Olson, Pamela Garcia-Filion

**Affiliations:** Division of Pediatric Endocrinology and Diabetes, Phoenix Children’s Hospital, 1919 E. Thomas Road, Phoenix, AZ 85016 USA; The Vision Center, Children’s Hospital Los Angeles, Los Angeles, CA USA

**Keywords:** Optic nerve hypoplasia, Septo-optic dysplasia, Panhypopituitarism, Hypopituitarism, Puberty

## Abstract

**Background:**

Endocrinologic abnormalities are a common co-morbidity in patients with optic nerve hypoplasia (ONH), however the impact on puberty is unknown. The purpose of this study was to examine rates of endocrine dysfunction and pubertal disturbances in a pediatric population of ONH.

**Methods:**

A retrospective chart review was conducted on a cohort of children with ONH between January 2005 and March 2013. Endocrine dysfunction was determined based on laboratory evidence of hormone deficiency or hormone replacement. Pubertal disturbances were characterized based on presence of micropenis, tanner staging, menarche and hormone replacement. Pituitary abnormalities were classified using MRI findings. Descriptive statistics were used, and comparisons between groups were performed using the chi-square test.

**Results:**

During the study period, 101 patients underwent an endocrine evaluation (median age: 2.3 years [0.76 – 6.5]). Hypopituitarism was present in 73% of patients with growth hormone deficiency (56%) and hypothyroidism (54%) being the most common. Pubertal disturbances (n = 19) were common; micropenis in 31% (13/42) of males and 2% with precocious puberty. Half of adolescents (n = 4/8) were diagnosed with gonadotropin deficiency. Patients with MRI pituitary abnormalities were more likely to have endocrine dysfunction than those without (p = 0.004). The sensitivity and specificity of MRI pituitary abnormalities for hypopituitarism was 54% and 92%, respectively.

**Conclusions:**

A significant proportion of children with ONH have endocrine dysfunction. The high frequency of pubertal disturbances in this study emphasizes the need for long-term monitoring of developing endocrinopathy. While pituitary gland abnormalities are a good predictor of endocrine dysfunction, a normal pituitary gland does not rule out endocrinopathy.

## Introduction

Optic nerve hypoplasia (ONH) is a congenital malformation that manifests as a spectrum disorder of visual impairment with cerebral malformations, hypopituitarism and developmental delay [1-3]. The prevalence of ONH is estimated at 1.73 per 10,000 children [4]. In the United States, ONH is a leading cause of legal blindness in children age 3 years or younger [5].

One of the most widely studied and recognized clinical association of ONH is hypopituitarism, ranging from 6% to 82% depending on the study design and patient sampling [6-13]. In a prospective clinical registry of young children with ONH, endocrinopathy affected 60-79% of children by age five years [10-12]. The most common hormone deficiency is growth hormone (GH) followed by central hypothyroidism [9,11]. Clinical management guidelines recommend long-term follow-up given reports of evolving central hypothyroidism [14], subclinical GH deficiency (paradoxical “growth without GH”) [10], and the association of untreated hypopituitarism with developmental delay and even death [12-15].

Pubertal disturbances are an understudied endocrinopathy in children with ONH. There have been more than a dozen cases of precocious puberty and/or gonadotropin deficiency reported [16-18] implicating pubertal disturbances as an additional risk. The unknown prevalence is likely due to past research being limited to prepubertal cases and absence of long-term follow-up into adolescence.

The purpose of this study was to retrospectively examine the prevalence of hypopituitarism in a clinical cohort of patients with ONH age 18 years and younger. The results of this study provide additional evidence of endocrinopathy risk throughout the age continuum in children with ONH.

## Methods

A retrospective study of patients with ONH was conducted on all patients evaluated in an endocrinology clinic at Phoenix Children’s Hospital between January 2005 and March 2013. All patients with a documented diagnosis of ONH or septo-optic dysplasia were included. Data abstracted from the medical record included patient demographics, anthropometric measures (height, weight, weight for length, body mass index), hormone test results, medications, and neuroradiographic findings from magnetic resonance imaging (MRI). Self-identified race was obtained based on medical record documentation with the following classifications: Caucasian, Hispanic, Native American, African-American, or other.

Subjects’ endocrine status was determined based on laboratory evidence of a hormone deficiency or current hormone replacement prior to clinic presentation. Laboratory test results were obtained when available. Patients were classified as GH deficient (GHD) based on subnormal growth factors [insulin-like growth factor-1 (IGF-1) and insulin-like growth factor binding protein-3 (IGFBP3)] levels below reference ranges, failed GH stimulation test with peak GH level < 10 ng/mL (using arginine, clonidine, or glucagon as stimulation agents), or hormone replacement. Central hypothyroidism was determined based on free thyroxine (T4) levels below reference ranges or hormone replacement. Adrenal insufficiency was determined based on fasting morning cortisol below reference ranges, subnormal cortisol response to adrenocorticotropic hormone (ACTH) stimulation test (peak < 18 μg/dL) or hormone replacement. Patients treated with desmopressin were classified as having diabetes insipidus.

Pubertal disturbance was ascertained based on presence of micropenis (stretched penile length 2.5 standard deviations below the mean), advanced or delayed tanner staging, premature or absence of menarche, and/or the need for testosterone or estrogen replacement. Precocious puberty was determined based on presence of secondary sexual characteristics prior to age eight in females and age nine in males with evidence of pubertal levels of gonadotropins, testosterone or estradiol, or history of treatment with a gonadotropin-releasing hormone (GnRH) agonist. Gonadotropin deficiency was based on absence of menarche in females by age 15 or no secondary sexual characteristics by age 13 in females and 14 in males.

### Data analysis

A descriptive analysis was conducted using Stata 13.0 (College Station, TX). Clinical and demographic findings were summarized using medians (interquartile range) and proportions. Comparisons between groups were performed using the chi-square test for categorical variables. Statistical significance was defined as an alpha of <0.05.

## Results

### Cohort characteristics

Data from 101 patients with ONH were available for review. Table 1 presents the patient characteristics for the study sample. The median age at the time of clinic presentation was 2.3 years (0.7, 6.5). For the overall cohort, the median height and weight z-scores were −1.3 (−2.2, −0.3) and −0.7 (−1.7, −0.6), respectively. Among subjects less than age two years (n = 47), the median weight for length z-score was 0.6 (−0.6, 1.3). The median body mass index (BMI) z-score, measurable in those age two years or older (n = 50), was 0.7 (−0.3, 1.5).

**Table 1:** **Characteristics of ONH cohort**

	**%**	**N**
**Gender**		
**Female**	58.4	59/101
**Male**	41.6	42/101
**Referral Source** ^**a**^		
**Pediatrician**	69.3	70/101
**Ophthalmologist**	14.9	15/101
**Neurologist**	5.9	6/101
**Other**	5.9	6/101
**Self-referral**	2	2/101
**Unknown**	2	2/101
**Race/Ethnicity** ^**b**^		
**Caucasian**	44.6	45/101
**Hispanic**	24.8	25/101
**Native American**	12.9	13/101
**Other**	7	7/101
**Not documented**	10.9	11/101
**Laterality of ONH**		
**Bilateral**	74.3	75/101
**Unilateral**	11.9	12/101
**Not documented**	13.9	14/101
**Brain Malformations** ^**c**^		
**Pituitary abnormalities**	44.1	26/59
**Absence of septum pellucidum**	46.8	36/77
**Corpus callosum hypoplasia**	34.7	25/72
**Cortical dysplasia**	16.9	14/83
**Schizencephaly**	10.9	9/83
**Hydrocephalus**	8.4	7/83
**Arachnoid cyst**	6.0	5/83
**Endocrinopathies** ^**d**^		
**Any endocrinopathy**	72.3	73/101
**GH deficiency**	62.0	57/92
**Hypothyroidism**	54.0	54/100
**Adrenal insufficiency**	51.1	47/92
**Diabetes insipidus**	27.7	28/101

^a^Some insurance plans only allow the primary pediatrician to order referrals.

^b^Based on documentation in the medical record.

^c^Total number of subjects varies based on available MRI reports.

^d^Total number of subjects varies based on available lab results.

### Endocrine evaluation

Seventy-two percent (n = 73/101) of patients had at least one endocrinopathy (Table 1) and 57% (n = 58/101) had multiple pituitary hormone deficiency (MPHD) (2 or more hormone deficiencies). Among subjects with GHD (62%), the median height SDS was −1.6 (−2.5, −0.7) compared to −0.45 (−1.6, 0.2) deemed GH sufficient (p = 0.001). There were three cases of isolated hypothyroidism, one case of isolated adrenal insufficiency and one case of isolated gonadotropin deficiency. All cases of diabetes insipidus (n = 28) co-existed with another pituitary deficiency: GHD (71%), hypothyroidism (86%) and adrenal insufficiency (82%).

At the initial endocrine visit [median age 2.3 years (0.7, 6.5)], thyroid hormone levels were measured in 82% (TSH: 82% and free T4: 98%), cortisol in 86%, and GH surrogates (IGF-1 and/or IGFBP3) in 89%. Seventy-one percent underwent serial free T4 testing after initial normal thyroid tests. Nearly all patients (>80%) underwent follow up testing for cortisol (repeat cortisol and/or cortisol provocative testing: 86%) and GH (repeat IGF’s and/or GH stimulation test: 83%). Pubertal hormone levels, in those younger than age 6 months or at pubertal age (n = 41), were measured in 42% for luteinizing hormone (LH)/follicle-stimulating hormone (FSH) and 35% for testosterone or estradiol.

### Pubertal disturbances

There were 15 subjects with a pubertal disturbance prior to pubertal age: two with precocious puberty and 13 (out of 42 males) with micropenis. Among males with micropenis, two were followed into a pubertal age; both were Tanner I at age 10 and 16 years. There was no association between presence of micropenis and MPHD (p = 0.72).

Among subjects at a pubertal age (n = 26), 24 had a Tanner stage evaluation. Nine females (out of 15) were Tanner II-V and six were Tanner I (age 8–9 years old). Five males (out of 9) were Tanner II-V and four were Tanner I (age 10–16 years old).

There were 15 girls at potential menarchal age (≥9 years); 40% had spontaneous menarche (median age of onset 12 years; 10.5 – 13 years). In those with MPHD, 66% (n = 4/6) had spontaneous menarche by age 15.

Eight patients (two males and six females) were followed into an age when gonadotropin deficiency could be assessed, with 50% (n = 4/8) being diagnosed. One male with MPHD was diagnosed with gonadotropin deficiency based on a Tanner I stage at age 16 years with low FSH, LH, and testosterone levels (started testosterone therapy). Three females were diagnosed with gonadotropin deficiency and all had MPHD. One female had menarche at age 14, but subsequently developed irregular periods in her late teen years; she had low estrogen and LH / FSH levels and began estrogen therapy. A second female did not achieve menarche by 15 with only Tanner II breast development and no pubic hair (started estrogen therapy). A third female failed to achieve menarche by age 15 years with Tanner II pubic hair, no breast development, and low LH/FSH levels (lost to follow-up).

### Brain malformations

Eighty-two patients underwent a MRI. Table 1 lists the findings documented in the MRI report. An absent septum pellucidum, present in 47%, was not associated with hypopituitarism (75% *versus* 76%; p = 0.951). The pituitary gland status, documented for 59 subjects, was abnormal in 25 cases. The frequency of pituitary gland abnormalities was similar for those with (54%) and without (63%) the septum pellucidum (p = 0.530). Pituitary gland abnormalities included an absent or hypoplastic pituitary gland in (n = 18), ectopic posterior pituitary (n = 13), absent or truncated stalk (n = 13), and absent posterior pituitary bright spot (n = 4). An ectopic posterior pituitary gland (stalk present in nine patients) was associated with DI in three cases (stalk present in two). A non-visualized posterior pituitary bright spot was an isolated finding for three subjects (no DI), and associated with an absent stalk in one subject (no DI).

Hypopituitarism manifested in 96% of patients with a reported pituitary gland malformation, compared to 64% of those with an intact pituitary gland (p = 0.004). The sensitivity and specificity of MRI pituitary abnormalities for hypopituitarism was 54% and 92%, respectively. The predictive values were 96% (positive) and 36% (negative).

## Discussion

The high prevalence of hypopituitarism in our cohort confirms previous reports of endocrinopathy in a majority of cases of ONH [2,9-12]. Overall, pubertal disturbances affected one-fifth of the cohort.

Pubertal disturbances in association with ONH was first described in 1978 in a female with precocious puberty [16]. Since then there have been 11 reported cases of precocious puberty in children with ONH (Table 2). In the present study, precocious puberty affected two patients (2%). The exact mechanism of precocious puberty in ONH is unknown. Huseman et al. [16] originally postulated that precocious puberty may be due to “decreased inhibitory inputs from higher centers of the central nervous system.” Since ONH is a neurodevelopmental abnormality, it is reasonable to assume there could be a disruption in inhibitory inputs or an increase in excitatory inputs to the hypothalamic GnRH neurons, leading to dysregulated GnRH secretion and precocious puberty. Hydrocephalus and arachnoid cysts, cerebral developmental abnormalities known to cause precocious puberty [19], were seen in seven and five patients in this study. However, neither patient with precocious puberty had these abnormalities. Future studies should examine the association of precocious puberty with these cerebral abnormalities.

**Table 2:** **Pubertal disturbances in ONH**

**Study**	**Sample size**	**Precocious puberty cases**	**Gonadotropin deficiency cases**	**Micropenis cases**
**Huseman, 1978** [16]	5	1	-	-
**Margalith, 1985 **[20]	17	1	2	-
**Hanna, 1989** [17]	13	1	4	-
**Siatkowski, 1997 **[21]	35	1	-	2
**Nanduri, 1999 **[18]	25	-	5	-
**Phillips, 2001 **[6]	67	-	-	3
**Reidl, 2002 **[22]	25	-	1	-
**Birkebaek, 2003 **[7]	55	6	2	-
**Haddad, 2005 **[9]	56	1	-	12
**Mohney, 2013 **[23]	19	1	-	-

Pubertal disturbances are linked to a disruption of the hypothalamic-pituitary-gonadal (HPG) axis. Ascertainment of pubertal hormone deficiencies is complicated by the limited time periods for measurement of pubertal hormone levels. Infants undergo a ‘mini-puberty’ during the first six months of life. If this early window is missed, pubertal hormones cannot be measured until an age when secondary sexual characteristics may occur naturally (≥8 in females, ≥9 in males). In males, micropenis is the most commonly reported sign of pubertal disturbances in ONH, present in one-third of males in this cohort. Since micropenis is not present in all cases of gonadotropin deficiency, future research should include follow up of male patients with ONH into adolescence to establish a diagnosis of gonadotropin deficiency.

Researchers posit gonadotropin deficiency is less common in patients with ONH and MPHD compared to other causes of MPHD, which may reflect migration of GnRH neurons from the nasal mucosa to the hypothalamus after the proposed insult in ONH [18,24]. In ONH, the pituitary gland is structurally intact in the majority of cases [6,11,13]. Formation of the anterior pituitary gland precedes development of the posterior pituitary, stalk and hypothalamus at seven weeks. Hypothalamic releasing factors are present around 10–14 weeks, with a functional hypothalamic-hypophyseal portal system by the 11th week. This complex network allows communication of hypothalamic releasing factors to their respective pituitary somatotrophs, thyrotrophs, corticotrophs, and gonadotrophs, stimulating the release of anterior pituitary hormones. Unlike other hypothalamic releasing factor neurons, GnRH neurons migrate from the olfactory placode to the hypothalamus by 10–14 weeks [25,26]. It is reasonable to assume that an insult to the hypothalamic-pituitary axis prior to this time period may result in preserved GnRH function.

Gonadotropin deficiency has been reported in multiple patients with ONH (Table 2), yet a prevalence has been difficult to establish as very few patients are followed into pubertal age. Nanduri et al. [18] reported gonadotropin deficiency in 42% of children with ONH that reached pubertal age. Our study found gonadotropin deficiency in 50% of those at pubertal age. In other hypothalamic-pituitary diseases (without ONH), gonadotropin deficiency is common: 96% in child-onset craniopharyngioma and 86% in child-onset hypopituitarism (mostly idiopathic) [27].

The lower prevalence of gonadotropin deficiency in ONH may support the theory of an intact HPG axis due to a later GnRH migration into the hypothalamus. However, if this was entirely true, then no cases of gonadotropin deficiency would be expected. A completely functional HPG axis is needed for normal pubertal development. Gonadotropin deficiency could be explained by an insult to the pituitary gland itself, resulting in abnormal gonadotroph development. Very few patients in our study of pubertal age had MRI reports available, so pituitary abnormalities could not be evaluated in relation to gonadotropin deficiency.

Expert opinion recommendations encourage a thorough and serial endocrine evaluation on all patients with ONH regardless of MRI findings owing to the high prevalence of hypopituitarism, variability in timing of onset, and evolving hypopituitarism [1,14]. However, there is debate about the use of imaging findings to direct endocrinologic testing in patients with ONH [28]. Phillips et al. [6] and Birkebaek et al. [7] were the first to suggest selective screening for hypopituitarism in patients with pituitary gland abnormalities. In some reports, the sensitivity and specificity of pituitary abnormalities on MRI ranged from 85% to 96% and 57% to 100%, respectively [6,7,13]. A prospective study of young children with ONH reported a high specificity (100%) and a low sensitivity of 7% [10]. In our cohort, pituitary gland abnormalities on MRI had a sensitivity of 54% and specificity of 92%. The low negative predictive value in the study by Ahmad et al. [10] (29%) and our study (36%) indicates a structurally intact pituitary gland is a poor predictor of normal endocrine function. High negative predictive values reported in other studies may reflect incomplete endocrine screening.

The HPG axis was evaluated in nearly half of our patients less than 6 months old or at pubertal age. Given our findings of a pubertal disturbance in 19% of subjects and 50% of pubertal patients diagnosed with gonadotropin deficiency, an evaluation of the HPG axis should be performed in all patients with ONH.

There were several limitations in our study. Similar to other reports using a single endocrine clinic cohort, our study was subject to selection bias and differential hormone testing. A complete endocrine evaluation was not performed in all patients and may have resulted in an underestimated prevalence of endocrinopathy. Ascertainment of hypopituitarism relied on clinical documentation, rather than systematic testing and standardized classification. For GHD, growth pattern data were not available for many of the patients, which affected confirmation of diagnosis in those on replacement therapy or with no anthropometrics prior to initiating therapy. MRI testing was performed at several institutions and imaging centers. Variability in the interpretation of MRI pituitary abnormalities between neuroradiologists may have led to an under or over-reporting of pituitary abnormalities.

## Conclusions

Among children with ONH, the prevalence of endocrinopathy is high including abnormalities in the HPG axis. Practitioners should be aware of the potential for precocious puberty throughout childhood and once patients reach a pubertal age, pubertal progression should be monitored annually. Evaluation of the HPG axis should be performed when patients fail to initiate puberty by an appropriate age (13 in females, 14 in males). Presence of micropenis, failure to progress through puberty normally, primary amenorrhea, or irregular menses would also warrant evaluation. Due to conflicting negative predictive values in evaluating MRI pituitary abnormalities in association with endocrinopathy, a normal pituitary gland on MRI should not exclude patients with ONH from an endocrine evaluation.

## Abbreviations

ASP: Absence of the Septum Pellucidum
FSH: Follicle-stimulating Hormone
GH: Growth Hormone
GHD: Growth Hormone Deficiency
GnRH: Gonadotropin-releasing Hormone
HPG: Hypothalamic-pituitary-gonadal
LH: Luteinizing Hormone
MRI: Magnetic Resonance Imaging
ONH: Optic Nerve Hypoplasia
T4: Thyroxine
